# On the Use of Pituitary Extract in Obstetrics

**Published:** 1914-01

**Authors:** 


On the Use of Pituitary Extract in Obstetrics. F. C. Harrison, B.A., M. B. Assistant in Pharmacology, University of Toronto. *The Canadian Practitioner and Review,* November, 1913.

Although the first use of pituitary in obstetrical practice was made in England, but few papers dealing with its action have been published in English, while in Europe it has been so extensively used that a study of the large number of papers and cases now published enables one to formulate, with considerable precision, rules for its employment. This is especially true after one has given it, with success and failure, in one's own experience. This forms the justification for this paper.

In 1895, Oliver and Schaefer, in their series of experiments with organ extracts, followed up their papers on the striking effects produced by the injection of extracts of the adrenal by showing that extracts of the pituitary also produced a rise in blood-pressure. In 1898, Howell showed that it was the posterior lobe which possessed this property. This was confirmed by Schaefer and Vincent, and in 1901 Schaefer and Magnus showed that the extract of the infundibular portion increased very markedly the flow of urine. At this point the matter rested until 1906, when Dale, in the course of some observations on the action of ergot, noted that the extract of pituitary brought about a marked contraction. This observation, however, was entirely lost sight of until Blair Bell and Hicks, led by some experiments which they had on hand, obtained from Dale some pituitary extract and produced with it marked contractions of the uterus in pregnant rabbits.

In consequence of the result obtained, Blair Bell was led to use it in some obstetrical cases. He presented his observations before the Liverpool Medical Institution, November, 1909, and his paper was published in December. In it he refers to its use in two cases of post-partum hemorrhage, and one case in which it was used in the expulsive stage of labor. In September, 1910, Aarons of Edinburgh read a paper at the International Congress in St. Petersburg, in which he reported success in its use in six cases of post partum hemorrhage.

Hahl and Malinowsky have recorded the variations in intrauterine pressure with a bag, after the method of Westermarck, and in two cases reported by Hahl, there was some rise in tone, the movements being shorter and stronger, at shorter intervals and with increased intrauterine pressure; and Malinowsky shows a good tracing of a uterine tetanus. In other cases no increase in tone occurred, but only of movements. Such tetani are, not uncommon with ergot preparations, but apparently much less so with pituitary. Cases have, however, occurred. In one of the cases published by Rieck a tetanus evidently occurred, and is very well described by him. When it took place after the second injection, which was given six hours after the first injection, the patient stated that she felt the individual pains, but the examining hand could detect no contractions or relaxations.

Lieven also reports a case in which the uterine tetanus was so marked that the child's heart-rate fell steadily to 82, and there was a marked passage of meconium. Spaeth reports a similar case. He was, however, so unfortunate as to see the child die. Seitz and Roemer have each stated that they have also seen tetanus occur. Hamm, too, in one case which was brought to him after the child was dead and the patient had been in labor for a considerable period of time, injected pituitary, and regular movements set in. These, however, died away, and a second injection was given, which was followed in seven minutes by a tetanic constriction which lasted eight minutes, then regular movements for one or two hours, which gradually died away. A third injection produced another tetanus of eleven minutes' duration, then regular movements; and the fourth, tetanus of seventeen minutes, followed by regular movements, which led to delivery. He states that he has frequently seen the fœtal heart-rate fall to 80; but in view of the rapidity of delivery in most cases, does not consider this dangerous. The above incident shows quite clearly, however, that the use of pituitary is not without some danger to the child, though danger to the mother seems very slight. In consequence, the fœtal heart sounds should be, if it is at all possible, kept constantly under observation. This danger seems undoubtedly greatest when the drug is administered during the first stage.

The majority of observers, and especially those with the larger series of cases, have found that for the production of abortion, pituitary alone is insufficient. We might quote in this connection Schiffmann, who, out of seven cases, had three complete failures. Hell, Fischer, Nagy, Hirsch, Voigt, Merkel, Sellheim and Trapl have all pronounced against its use for this purpose.

Nor has it been of any great service in initiating premature labor. Trapl, Schiffman, Fischer, Nagy, Hirsch, Voigt, Foges and Hofstätter, Merkel and Sellheim may be quoted in support of this statement. Nevertheless, successful cases have been observed in which one or two injections sufficed; and in the production of both abortion and premature labor, it has proved in almost all cases a useful aid to other methods, such as dilatation by mechanical means.

Practically all observers are unanimous in declaring that it is of the greatest value in overcoming uterine weakness supervening after dilatation of the soft parts, and during the expulsive stage of labor. The effect, as a rule, is very prompt. The pains set in with great vigor in fifteen to twenty minutes or less, and are strong, rapidly leading to delivery; sometimes in a few minutes, and frequently within the hour. As mentioned above, pituitary has been used in at least 1,650 cases, but it is difficult to estimate in how many of these cases it was given during this phase of labor. Not more than a dozen failures during this stage are recorded. The total number of complete failures reported is less than fifty, and these failures are largely amongst those cases in which it was used for production of abortion or premature labor, without other means being employed, or post partum. Several observers have found that it produces movements that are so rhythmical and strong as to be of the greatest value in converting abnormal into normal positions. There is, of course, no general agreement as to what constitutes failure; and as details in all the longer series of cases are not given, it is impossible to express the results recorded in the literature in a more exact fashion. Hofbauer, in his last forty cases, reports no failures; Cahn, out of eighty-seven cases, three; Aubert, 15 per cent. of failures; Foges and Hofstätter, out of sixty-three cases, three failures. These figures seem very typical; but it must be noted that other observers, with as large a series or larger, say nothing of failure.

The author cites four personal cases and gives the following conclusions, as well as a very complete libliography:

1. Pituitary is of great value in cases of weakness in uterine movements after the soft parts are well dilated. Failure in these cases is rare, probably less than 1 per cent. The later in labor, but before delivery, the more striking the effect. The danger to the child and mother is very slight.

2. As an addition to same mechanical method, e.g., the Champetier de Ribes' bag, it is of great value in bringing on premature labor or abortion. In the former case it may be sufficient in itself, but there is some risk of tetanus of the cervix, or of the uterus, especially when repeated injections are required.

3. For delivery of the placenta its use is accompanied by the danger of tetanus uteri and retention.

4. In post-partumhæmorrhage a considerable percentage of failures may be expected.

When a need for a uterine stimulant arises in cases conforming to the above indications, I believe that pituitary is of the greatest value, and will act as in cases 1 and 2, which are typical of others in my experience.

I desire to express thanks to Dr. V. E. Henderson for the use of his laboratory, and invaluable assistance and criticism in preparing this paper.

